# Can Popular High-Intensity Interval Training (HIIT) Models Lead to Impossible Training Sessions?

**DOI:** 10.3390/sports10010010

**Published:** 2022-01-06

**Authors:** Jérémy Briand, Jonathan Tremblay, Guy Thibault

**Affiliations:** 1 Institut National du Sport du Québec, 4141 Avenue Pierre-De-Coubertin, Montreal, QC H1V 3N7, Canada; jeremy.briand@umontreal.ca (J.B.); gthibault@insquebec.org (G.T.); 2 École de Kinésiologie et des Sciences de l’Activité Physique, Faculté de Médecine, Université de Montréal, 2100 Boulevard Édouard-Montpetit, Montreal, QC H3T 1J4, Canada

**Keywords:** critical power, anaerobic reserve, modeling, exercise prescription, intermittent exercise

## Abstract

High-Intensity Interval Training (HIIT) is a time-efficient training method suggested to improve health and fitness for the clinical population, healthy subjects, and athletes. Many parameters can impact the difficulty of HIIT sessions. This study aims to highlight and explain, through logical deductions, some limitations of the Skiba and Coggan models, widely used to prescribe HIIT sessions in cycling. We simulated 6198 different HIIT training sessions leading to exhaustion, according to the Skiba and Coggan-Modified (modification of the Coggan model with the introduction of an exhaustion criterion) models, for three fictitious athlete profiles (Time-Trialist, All-Rounder, Sprinter). The simulation revealed impossible sessions (i.e., requiring athletes to surpass their maximal power output over the exercise interval duration), characterized by a few short exercise intervals, performed in the severe and extreme intensity domains, alternating with long recovery bouts. The fraction of impossible sessions depends on the athlete profile and ranges between 4.4 and 22.9% for the Skiba model and 0.6 and 3.2% for the Coggan-Modified model. For practitioners using these HIIT models, this study highlights the importance of understanding these models’ inherent limitations and mathematical assumptions to draw adequate conclusions from their use to prescribe HIIT sessions.

## 1. Introduction

Interval training, often referred to as high-intensity interval training (HIIT), is a training method where, in a single session, exercise bouts performed at high intensity are interspersed with periods of active or passive recovery, both lasting from a few seconds to a few minutes [1,2]. Many studies have shown that HIIT can be a time-efficient and enjoyable method to improve health, fitness, and sports performance for clinical populations, healthy subjects, and athletes [3,4,5]. Although HIIT is widely used in many sports [6], the prescription of HIIT sessions involves several parameters such as the number of sets and repetitions per set, the duration and intensity of the high-intensity and recovery intervals, etc. Every parameter can impact the overall difficulty level of the session. For any given individual, it is therefore hard to anticipate if the prescribed session will lead to the desired training load [7]. There exists a variety of models to predict and better understand performance and fatigue over continuous exercise: the two-parameter (or hyperbolic) critical power model [8], the three-parameter critical power model [9], the Péronnet–Thibault model [10], and the Omni power-duration model [11]. The two-parameter critical power model was adapted to intermittent exercise and improved through various iterations [9,12,13,14,15]. Other models, by Coggan [16], Hayes [17], and Purdy [18], can assist practitioners in the prescription and the analysis of HIIT sessions. In cycling specifically, two models stand out. The Skiba model [14,15], widely referred to in the scientific literature, and the Coggan model [16], commonly used in the field, in fact by millions of users, in part through commercial apps such as TrainingPeaks (www.trainingpeaks.com, accessed on 20 November 2021). The main goal in both models is to assess an individual’s residual fatigue or capacity while performing repeated high-intensity exercise bouts, thus quantifying the relative training load or difficulty level of any HIIT session, and planning accordingly.

### 1.1. The Skiba Model

This HIIT model is based on the Monod and Scherer hyperbolic model [8] for continuous exercise, developed more than half a century ago. The model takes the form of the following equation:

(1) $$P=\frac{W^{\prime }}{t_{lim}}+CP$$

where $t_{lim}$ is a given time to exhaustion, and *P* is the maximal average power sustained over that duration.

Two key performance indices are derived from the Monod and Scherer model: (1) the critical power (*CP*), which is the power corresponding to the asymptote of the hyperbolic relationship between power and exercise duration, and (2) the *W*′, often assimilated to the anaerobic energy reserve or the amount of work that can be completed above *CP* [19,20]. The parameters are found by performing a linear fit of the athlete’s work produced (product of power and duration) over various exercise trials (or competitions) of durations typically ranging between 2 and 15 min [21,22]. The slope of the linear fit corresponds to the *CP*, and the zero intercept to the *W*′. The *CP* is sometimes considered a boundary between the heavy and severe intensity domains.

The Skiba HIIT model puts forward that exercise at an intensity inferior to the *CP* is performed in a steady-state and could theoretically be sustained indefinitely [23]. This model posits that work performed above the *CP* depletes the *W*′ at a rate proportional to the difference between the actual power output and the *CP*. It also asserts that when the *W′* is completely depleted (*W′_balance_* = 0), the athlete has reached exhaustion [19] and cannot pursue the effort without prior recovery.

Different attempts were made to adapt the Monod and Scherrer continuous exercise critical power model to intermittent exercise and track the *W′_balance_* along the repeated exercise and recovery bouts [9,12]. In the Skiba model latest iteration [15], work performed below the *CP* allows replenishing *W′* curvilinearly, at a rate proportional to the difference between the actual power output during recovery and the *CP*. The following set of equations describes the *W′* depletion and its recovery. The rate of *W′* depletion is always proportional to the difference between the power output (P) and the *CP*. Thus,

(2) $$\frac{d\;W^{\prime }\left(t\right)}{dt}=-\left(P-CP\right)$$

Solving the differential equation (Equation (2)) for the depletion:

(3) $$W^{\prime }\left(t_{i}\right)=W^{\prime }\left(t_{i-1}\right)-\left(P_{i}-CP\right)\cdot \left(t_{i\;}-t_{i-1}\right)$$

As for the replenishment, the differential equation differs slightly. The replenishment is still proportional to the difference between *P* and the *CP*. However, it is also determined by the amount of *W′* expended from the initial value ($W_{0}^{\prime }$). The *W′* reconstitution differential equation is:

(4) $$\frac{d\;W^{\prime }\left(t\right)}{dt}=\left(\;1-\frac{W^{\prime }\left(t\right)}{W_{0}^{\prime }}\right)\cdot \left(CP-P\right)$$

Solving the *W′* reconstitution differential equation (Equation (4)):

(5) $$W^{\prime }\left(t_{i}\right)=W_{0}^{\prime }-\left[W_{0}^{\prime }-W^{\prime }\left(t_{i-1}\right)\right]\cdot e^{\left[\frac{P_{i}-CP}{W_{0}^{\prime }}\;\left(t_{i}-t_{i-1}\right)\right]}$$

### 1.2. The Coggan Model

A second HIIT model commonly used in cycling is the Coggan model [16]. It starts with the principle that work (product of power and duration) is a poor indicator of training load; it introduces new metrics reflecting the curvilinear relationship between power output and the physiological responses. For any HIIT session, it allows to compute a Normalised Power ($P_{N}$), an Intensity Factor (IF), and a Training Stress Score (TSS) [16]. The $P_{N}$ is calculated by an algorithm which (1) takes the average power output over 30 s rolling average windows, (2) raises the average power of each window to the fourth power, (3) computes the mean of the values obtained at step 2, and (4) takes the fourth root of that mean. The IF is a dimensionless value proposed by Coggan to put in perspective the $P_{N}$ of the session with respect to the athlete’s highest average power output sustained over an hour (referred to as the Functional Threshold Power, or FTP):

(6) $$IF=\frac{P_{N}}{FTP}$$

TSS is a metric used to quantify training load. It is computed from the $P_{N}$ and the IF:

(7) $$TSS=\frac{T\cdot IF\cdot P_{N}}{FTP\cdot 3600}$$

where *T* is the total duration of the session in seconds.

### 1.3. Practical and Theoretical Value of the Models

On the one hand, the Skiba model and the *CP* concept are commonly referred to in the scientific literature. The *CP* models are considered simple, robust, and promising [20,24]. The parameters (*CP* and *W′*) are presumed to be associated with physiological responses to exercise [20,25,26,27]. On the other hand, the *CP* concept is also widely criticized and referred to as a purely mathematical artifact with very few practical applications [28,29]. The Skiba model is yet to be adopted by endurance coaches [25]. The *W′* reconstitution requires further work and individual adjustments [19,25,30,31]. At the foundation of the Skiba HIIT model, the continuous exercise hyperbolic model relies on a specific set of assumptions [25] restricting its validity to limited intensity and duration windows [32]. We suspect that the limitations of the hyperbolic model propagate to the Skiba HIIT model and confine its validity to certain intensity windows.

The Coggan model is widely used in practice but has obvious logical limits. For instance, the IF and TSS are derived using a single performance, the FTP, which does not account for the global athlete performance profile. Two athletes may have the same FTP, but one could produce higher power outputs on shorter effort durations, while the other, on longer effort durations. By relying on a single metric (FTP), we suspect the model can provide different outcomes depending on the athlete’s global power profile.

The aims of this paper are to (1) show, through logical deductions and computer simulations, the limits of the Skiba and Coggan models in the prescription and analysis of intermittent efforts; (2) put forward explanations as to why these logical issues occur; and (3) discuss how these limits affect the practical applications of the models.

## 2. Materials and Methods

The feasibility of an extensive range of simulated HIIT sessions was assessed for three fictitious athletes.

### 2.1. Fictitious Athletes’ Profiles

In his book, Coggan [16] provides examples of the power profile distribution for different types of cyclists. The Péronnet–Thibault [10] continuous exercise model enables the computation of an athlete’s complete power-duration profile given three inputs: the anaerobic capacity, maximal aerobic power (MAP), and endurance. Based on different power-duration distribution examples put forward by Coggan [16], we defined three profiles: (1) Time-Trialist (lower anaerobic capacity, average MAP and higher endurance), (2) All-Rounder (average anaerobic capacity, MAP and endurance), (3) Sprinter (higher anaerobic capacity, lower MAP and lower endurance) The All-Rounder is meant as a baseline profile, while the Sprinter displays a higher anaerobic capacity and, the Time-Trialist, a higher endurance level. The various profiles allowed to assess the influence of the athletes’ anaerobic capacity and endurance on the model’s outputs. Table 1 provides the anaerobic capacity, MAP, and endurance values used in the Péronnet–Thibault model to generate each profile, as well as their *CP* and *W′* values. For simplicity, we arbitrarily chose male athletes (70 kg body mass), with basal metabolic rates of 1.2 W/kg, and a cycling gross efficiency of 20% [33]. We also simplify the MAP as the athlete’s best power performance over 5 min [2,34]. The inputs and outputs of the Péronnet–Thibault model are absolute metabolic powers produced by the athletes to which the cycling gross efficiency of 20% has to be applied to obtain the usual mechanical power (as measured by a power meter). Table 2 presents each athlete’s best mechanical power performance (in W) over a range of durations (between 1 s and 4 h).

### 2.2. Combinations of HIIT Parameters

For each fictitious athlete profile, we generated several HIIT sessions over a large spectrum of possibilities. To define this spectrum, variations in the values for each HIIT session parameter were made as such: interval durations between 15 s and 5 min, by increments of 15 s; a number of repetitions between 2 and 20, by increments of one repetition; rest durations between 15 s and 5 min, by increments of 15 s. By testing every combination of the above parameters, we obtained 6198 combinations, after filtering out the combinations with a total session duration of less than 30 s, required to compute the $P_{N}$, and longer than 90 min, which are rarely performed in practice [7]. The rest intensity was arbitrarily set as 50% of the athlete’s MAP, as it is an intensity that could reasonably be adopted naturally by the athletes for recovery, and frequently used in studies on active recovery [35,36,37]. The interval exercise intensity was set such that the last repetition led to exhaustion, according to each model (Skiba and Coggan).

### 2.3. Exhaustion, According to the Skiba Model

According to the Skiba model, the work intensity was adjusted such that exhaustion occurs when the *W′* is completely depleted at the end of each of the 6198 sessions [25].

### 2.4. Exhaustion, According to the Coggan Model

Coggan does not refer to exhaustion in his book [16]. To appreciate exhaustion through the Coggan model, we made a subtle logical modification to it, that we will refer to as the Coggan-Modified model. The interpretation puts in perspective the HIIT session TSS by comparing it with the maximal possible TSS over the session duration.

Rearranging equation (Equation (7)), we obtain:

(8) $$TSS=\frac{T\cdot IF\cdot P_{N}}{FTP\cdot 3600}={\left(\frac{P_{N}}{FTP}\right)}^{2}\cdot \frac{T}{3600}$$

Then, if we consider a maximal effort over duration *T*:

(9) $$TSS_{max}={\left(\frac{P_{max}}{FTP}\right)}^{2}\cdot \frac{T}{3600}$$

where $P_{max}$ corresponds to the maximal power output produced by the athlete over a duration *T*.

If the athlete were to do a HIIT session of duration *T*, leading to exhaustion, the TSS of that session would be maximal ($TSS_{max}$). Therefore, we obtain the following:

(10) $$TSS_{max}={\left(\frac{P_{N}}{FTP}\right)}^{2}\cdot \frac{T}{3600}$$

(11) $${\left(\frac{P_{N}}{FTP}\right)}^{2}\cdot \frac{T}{3600}={\left(\frac{P_{max}}{FTP}\right)}^{2}\cdot \frac{T}{3600}$$

(12) $${\left(\frac{P_{N}}{FTP}\right)}^{2}={\left(\frac{P_{max}}{FTP}\right)}^{2}$$

(13) $$P_{N}=P_{max}$$

To lead to exhaustion, the $P_{N}$ of the session must match the athlete’s maximal power output over the session duration. Given the 6198 combinations of HIIT parameters, we set the work intensity such that the $P_{N}$ matches $P_{max}$ over the session duration.

We thus end up with two sets of 6198 HIIT sessions for each fictitious athlete profile: a first set leading to exhaustion according to the Skiba model, and a second set according to the Coggan-Modified model.

## 3. Results

By examining the simulated HIIT sessions, we encountered impossible sessions, i.e., sessions for which the work intensity derived from the theoretical model (Skiba or Coggan-Modified) would require the athlete to surpass, on every effort interval, his maximal power output over the work interval duration.

**Figure 1:**
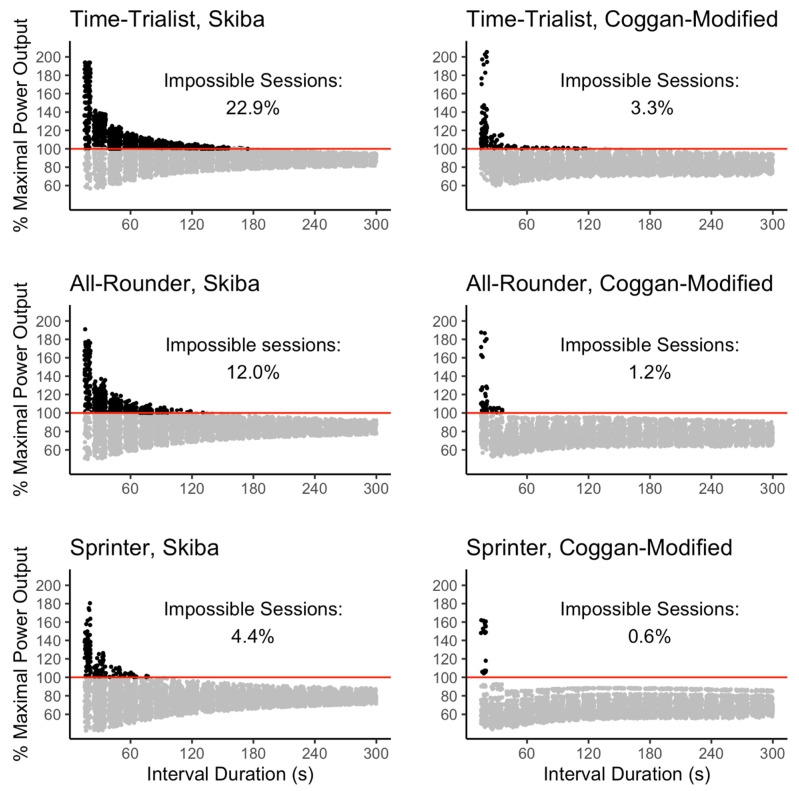
Simulated HIIT sessions using the Skiba and Coggan-Modified models for each fictitious athlete profile. The *x*-axis refers to the interval duration of each training session, and the *y*-axis to the power output expressed as a percentage of the athlete’s maximal power output over the work interval duration. The black dots above the solid line correspond to sessions impossible to realize in practice. These sessions require, on every work interval, to surpass the maximal power output over the work interval duration. The relative percentage of the 6198 HIIT impossible sessions in practice for each model and each athlete profile is also reported on each respective graph.

Figure 1 presents six graphs showing each HIIT session simulated for the Skiba and the Coggan-Modified models and each respective athlete profile. For each profile, both models lead to sessions logically impossible to complete, requiring athletes to better their personal best performance at each exercise interval. Other HIIT sessions amongst the ones simulated are likely impossible to realize, even though they do not imply per se that the athlete must surpass his best performance at every repetition. Thus far, our analysis does not identify such sessions and is limited to sessions breaking the athletes’ absolute limit, introducing a logical contradiction in the model.

The graphs in Figure 1 also report the relative percentage of the 6198 HIIT sessions that are logically impossible to realize for each model and each athlete profile. For both models, the percentage of impossible sessions is higher for the Time-Trialist. The All-Rounder profile has a lower percentage of impossible sessions than the Time-Trialist but higher than the Sprinter. The Sprinter is the profile displaying the lowest percentage of impossible sessions. The relative percentage of impossible sessions is always higher for the Skiba model than the Coggan-Modified model.

**Figure 2:**
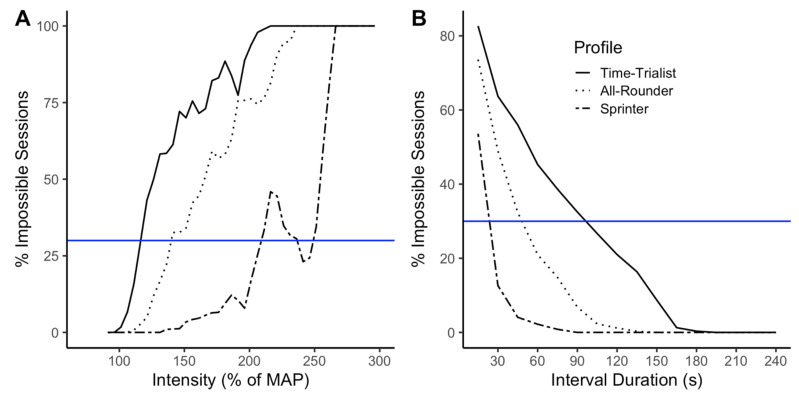

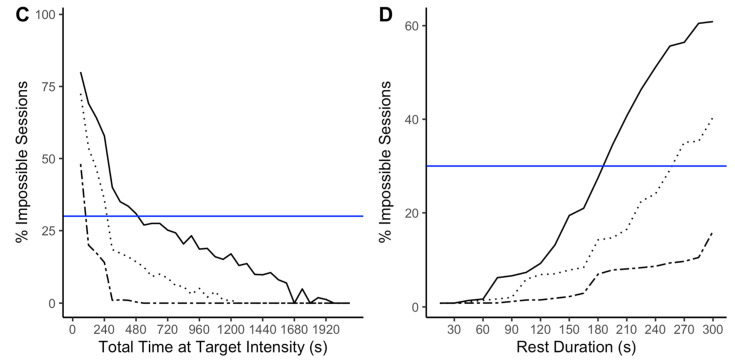
Using the Skiba model, percentage of impossible sessions within subsets of (**A**) Prescribed work intervals power output expressed in percent of the MAP, (**B**) Interval durations, (**C**) Total time spent at target intensity, and (**D**) Rest durations. Each athlete profile is represented by line type (Time-Trialist: solid line, All-Rounder: dotted line, Sprinter: dashed line). For reference, the blue line corresponds to 30% of impossible sessions.

**Figure 3:**
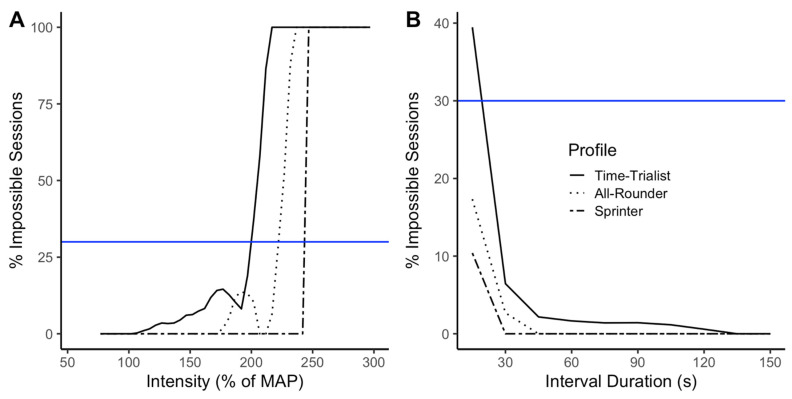

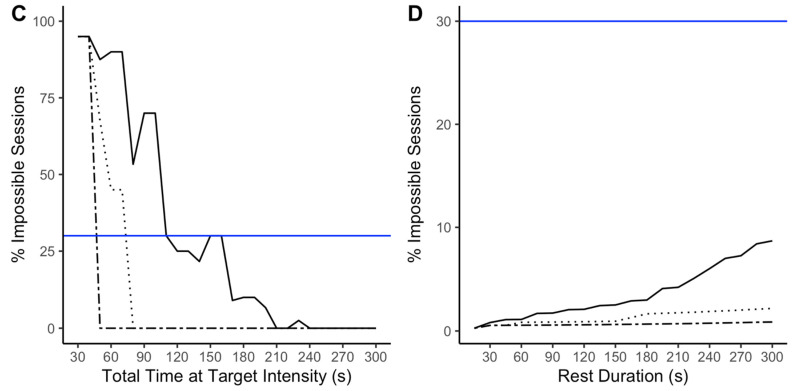
Using the Coggan-Modified model, percentage of impossible sessions within subsets of (**A**) Prescribed work intervals power output expressed in percentage of the MAP, (**B**) Interval durations, (**C**) Total time spent at target intensity, and (**D**) Rest durations. Each athlete profile is represented by a different line type (Time-Trialist: solid line, All-Rounder: dotted line, Sprinter: dashed line). For reference, the blue line corresponds to 30% of impossible sessions.

The HIIT parameters influence the occurrence of impossible sessions. Figure 2 (Skiba model) and Figure 3 (Coggan-Modified model) show the impossible sessions over subsets sharing the same HIIT parameters. The parameters studied are (A) the models’ prescribed work interval power output in percent of the MAP, (B) interval duration, (C) total time spent at target power output (product of the number of repetitions and interval duration), and D) rest duration. The curves of the graphs in Figure 2 and Figure 3 (especially for Figure 2A,C and Figure 3A,C) are not necessarily smooth, which will be discussed in the next section. However, looking at the general trends of each curve, we observe that impossible sessions are characterized by a small number of short intervals at high target intensity, in what is typically referred to as the severe and extreme intensity domains [32], with long recovery periods in between the efforts. For the same relative power output, interval duration, total time spent at the target intensity or rest duration, the profile corresponding with the highest level of endurance (Time-Trialist) is subject to a higher percentage of impossible sessions than the athlete with a high anaerobic capacity (Sprinter). Similarly, the Skiba model produces a greater percentage of impossible sessions than the Coggan-Modified model for the same relative power output, interval duration, time spent at target intensity, or rest duration. Figure 2A and Figure 3A report the intensity as prescribed by the models. As expected, the prescription goes to very high intensities that are impossible to reach for the athletes. Nonetheless, it is important to notice that HIIT sessions prescribed at intensities ranging between 100 and 150% of the MAP, which are commonly prescribed by sports practitioners [7], could lead to impossible sessions for every athlete profile if prescribed using the Skiba model, and only the Time-Trialist profile, if prescribed using the Coggan-Modified model.

## 4. Discussion

This paper aimed to identify potential limits in the Skiba and Coggan models. Simulating fictitious athlete profiles and HIIT sessions shows that logical contradictions occur. The impossible sessions are characterized by a small number of short effort bouts at power outputs in the severe and extreme intensity domains, and long rest duration in between the efforts. This section will discuss why these contradictions are observed and how they impact the models’ practical applications.

### 4.1. Variations in the Percentage of Impossible Sessions

In Figure 2A and Figure 3A, the general tendency is that the percentage of impossible sessions increases as the prescribed intensity goes up. However, drops in the percentage of impossible sessions can be observed. The most significant drops observed correspond to sessions composed of 15 s work interval duration, which, in some cases, are possible to realize at the prescribed intensity, hence the observed decrease in the number of observed impossible sessions. Similarly, in Figure 2C and Figure 3C, the general tendency is a lower percentage of impossible sessions as time spent at the target intensity increases. However, we observe increases in the percentage of impossible sessions at certain target intensities, due to sessions sharing the same work interval duration and number of repetitions, but different rest durations. With greater rest duration, a higher work intensity is required to reach exhaustion, which can sometimes lead to impossible HIIT session configurations. These observations highlight the complexity of HIIT training modeling and the influence of every parameter on the session. It also puts forward some limitations in our simulation. We can hypothesize that simulating more combinations, for example using smaller increments (ex: 1 s instead of 15 s for the interval duration), would lead to much smoother curves. Nonetheless, the analysis remains relevant showing that impossible sessions do occur for specific parameters combinations and at intensities slightly above MAP, for both the Skiba and Coggan-Modified models.

### 4.2. Limitations of the Skiba Model

Built on the Monod and Scherrer hyperbolic relationship, the Skiba model relies on six assumptions [25]: (1) the *CP* is a boundary between the heavy and severe intensity domains, (2) work performed at intensities below this boundary can be sustained indefinitely and is supplied by aerobic mechanisms only, (3) work performed above the *CP* are soliciting anaerobic mechanisms, which is limited and expressed as a reservoir (*W′*), (4) exhaustion is reached when *W′* is depleted, (5) as duration tends to zero, the theoretical power output that the athlete could generate is infinite, and (6) movement efficiency remains constant. These assumptions imply that work performed below the *CP* can be sustained indefinitely, which is “physiologically flawed” [28]. In fact, studies of time to exhaustion (TTE) at *CP* report mixed results ranging from 20–40 min [38] up to over an hour [39]. Recently, a study showed that the *CP* was reduced after 80 min of heavy-intensity work, but that carbohydrate supplementation could attenuate this reduction [40]. Studies have also shown TTE trials to provide a potential learning effect [39]. These studies raise questions on the variability of the *CP* concept which has also been referred to as a purely mathematical artifact highly influenced by the duration of the exercise bout used to compute *CP* [28]. Another problem of the *CP* model is that as the exercise duration tends to zero, it predicts that the athlete could produce an infinite power output. The Morton 3-parameter continuous exercise model [41] has been designed to correct this specific limitation. The Péronnet–Thibault model [10] is another example of a more complex continuous exercise model that corrects some of the assumptions of the hyperbolic model. Nevertheless, the hyperbolic model remains the foundation of the Skiba model. As highlighted by Chorley and Lamb [25], the cycling training prescription and analysis commonly made based on the Skiba model relies primarily on cases falling within the model assumptions. The logical contradictions observed in our simulations occur specifically in cases falling outside the model assumptions for an interval duration of fewer than 3 min. While these limitations are well-acknowledged for the continuous hyperbolic model [32], we provide mathematical evidence that they propagate to the Skiba intermittent exercise model and affect the prescription and analysis of intermittent efforts in the severe and extreme intensity domain.

The impossible sessions observed with the Skiba model occur in the severe and extreme intensity domains because the athletes’ capacities are overestimated by the hyperbolic model in that specific intensity domain. The model assumes more *W′* available than in reality, consistent with observations suggesting that the *W′* is not entirely depleted after the athlete reached exhaustion [31]. It implies that the athlete would have to produce an “impossible” effort in practice to deplete the *W′* completely. For athlete profiles with greater endurance, such as the Time-Trialist, their limited capacities on short exercise duration increase this overestimation and lead to more logical contradictions.

### 4.3. Limitations of the Coggan Model

The $P_{N}$ algorithm, presented in Coggan’s book [16], is suggested to reflect the curvilinear relationship between the work intensity and the physiological responses. At a high relative power output, where impossible sessions are observed, for the session to lead to exhaustion, the power output that must be sustained for each interval according to the Coggan model is higher than the athletes’ best performance over the interval duration. Since the anaerobic metabolism is predominant for exercise intensity in the severe and extreme intensity domain, we believe the $P_{N}$ algorithm could be adapted in order to correct logical contradictions, because the physiological mechanisms at play in these intensity domains are different, affecting the functional relationship between exercise intensity and the physiological responses. Our simulations also show that the athlete’s endurance influences the number of impossible sessions. There might not be a universal power (in the case of $P_{N}$, the fourth power) to represent curvilinear relationships between the physiological responses and intensity of every individual. An athlete with greater endurance (Time-Trialist, for example) might need a higher exponent to adequately represent the physiological responses induced by supra-maximal intensity. Athletes with a high anaerobic capacity (such as the Sprinter) would more easily “match” the session $P_{N}$ to their maximal power output over the session’s duration, due to their lower endurance level and their ability to produce a higher power output over short duration intervals.

As shown in Figure 3, the Coggan-Modified model globally displays less logical contradiction than the Skiba model. The Coggan model, as presented in the book [16], is somewhat limited as it only allows for relative comparisons (presented in tables) of IF and TSS. All the relative indices presented in Coggan’s book rely on a single performance metric, the FTP. The Coggan-Modified model introduces a subtle modification bringing an added dimension to it. By comparing the TSS of the training session with the athlete’s maximal TSS over the session duration, the Coggan-Modified model does not rely on the FTP anymore but on the athlete’s power profile as a whole. We think this added dimension is likely one of the reasons why the Coggan-Modified model appears less limited than the Skiba model in our simulations.

### 4.4. Applicability of the Models

Impossible sessions, in our analysis, imply that to reach exhaustion according to the Skiba and Coggan-Modified models, athletes would have to surpass their maximal power output over the work interval duration. In practice, it implies that HIIT sessions prescribed using the Skiba model or the Coggan-Modified model, within the same intensity, interval duration, total time at target intensity, and rest duration ranges as the observed impossible sessions, would not lead to the desired or expected training response. Both Skiba and Coggan models were initially derived from continuous exercise models and adapted for intermittent exercise. Our simulations suggest that the limitations of the Monod and Scherer hyperbolic model propagate through the Skiba model. Using a model which relies on the athletes’ global performance profile, such as the Coggan-Modified model, limits the observed logical contradiction. Traditionally, cyclists (and athletes in other endurance sports) spend a significant fraction of their training volume around a “tempo” intensity or near the lactate threshold [42,43,44], which stands at ~80–90% of their MAP. The Skiba and Coggan-Modified models do not lead to impossible training sessions in that intensity domain, but the selected intensity can nevertheless affect the physiological responses and adaptations associated with the session [7,42]. Numerous studies suggest other intensity domains to be of interest [7,45]. Indeed, HIIT performed within the severe and extreme intensity domains has gained popularity over the last years, notably through the contribution of Tabata [46,47], Gibala [48,49,50,51], and Seiler [52,53]. Moreover, a cycling race can be seen as a complex, stochastic form of HIIT, composed of inevitable supra-maximal efforts. The Coggan and Skiba models also aim to analyze race data to assist pacing and race strategies [16,27]. In short, the training and competitive demands now require the analysis and prescription of HIIT in the severe and extreme intensity domains, and both the Skiba and Coggan-Modified models have been shown to have limitations for that purpose.

### 4.5. Limitations

The analysis approach used in this paper is somewhat unconventional. It was mainly motivated by the challenge that constitutes reliable data derived from athletes’ power profiles. Not only accessing a large sample of real training data is challenging but defining an athlete’s performance profile is also puzzling. It requires determining whether each point in the power profile represents the athletes’ current fitness and if the test was really maximal and performed to exhaustion. To avoid those complications, a simulation approach was preferred, which, for simplicity, is limited to a restricted number of profiles that could be attributed to elite-level male athletes. The observed outcomes would likely be similar with recreational athletes since it is performed relative to the athlete’s MAP. In a sense, the simulation resembles a proof by contradiction in mathematics, where only a single case figure serves to demonstrate a contradiction of the initial statement (in this case, Skiba and Coggan models). Simulating over a greater variety of power profiles and larger combinations of sessions would likely give more insights and help define the extent of the models limits. The intensity of the recovery bouts was arbitrarily set at 50% of the athlete’s MAP. However, there is evidence in the literature that passive recovery leads to higher work interval power output or velocity [54]. Simulating over various recovery intensities would provide additional information on the extents of the models’ limitations as lower recovery intensities would necessarily lead to more logical contradictions from the models, because of the increased work intensity. As a final note, being derived from continuous exercise models, both Skiba and Coggan models potentially neglect factors that could influence the performance over intermittent efforts, such as the athlete’s ability to repeat high-intensity efforts, motivation, or residual fatigue at the beginning of the session, and potential learning effects induced by performing numerous HIIT sessions.

### 4.6. Future Perspective

Other HIIT models exist to assist practitioners in the prescription of HIIT training. The Purdy model [18], developed for running, does not currently have the same commercial impact as the Coggan model nor generate the same interest in the scientific literature. It is built along a train of thought more specific to the sport of running, where the intensity of work intervals is set as a percentage of the race pace over a target distance rather than using any physiological marker [1]. In doing so, the model never requires athletes to execute work intervals at an intensity superior to their personal best. Adapting the Purdy model to other cyclic sports, such as cycling, and making it more robust and polyvalent could greatly benefit the practitioners’ ability to prescribe HIIT training in cycling.

An obvious solution to the limits of the Skiba model could be to adapt the Péronnet–Thibault model [10] and Morton model [41], which relax the assumptions of the Monod and Scherrer model, to intermittent efforts.

To our knowledge, the latest iteration of the Skiba [15] model has yet to be validated. Although three validations have been attempted on the first iteration [55,56,57], many studies conclude that to this day, the reconstitution of W′ requires further research [19,25,30], the model is subject to inter-athlete variability and needs individual adjustments [19,25,30]. On the other hand, there appears to be only one form of validation of the Coggan model: a study by Wallace et al. [58] measuring the training load associated with performance on a 1500 m run using Training-Impulse (TRIMP) [59], Session-Rating of Perceived Exertion (sRPE) [60], and TSS. The three training load indices were shown to be highly correlated with the 1500 m running performance [58]. In order to be useful for practitioners, models need to be valid and reliable, and more data is needed to assess the current models.

## 5. Conclusions

Through a set of logical deductions and simulations, this study shows the limitations of two models commonly used to prescribe and analyze HIIT sessions in cycling. The main takeaway is that both the Skiba and the Coggan models are limited in analyzing and prescribing HIIT in the severe and extreme intensity domains, especially for sessions with a low number of short interval durations and long recovery. When working with such models, practitioners should be aware of their limitations for HIIT prescription to remain relevant, and draw adequate conclusions from their analysis.

## Figures and Tables

**Table 1:** Estimated physiological characteristics of each fictitious athlete. The endurance, anaerobic capacity, and MAP were used as parameters in the Péronnet–Thibault (1989) continuous exercise model to obtain each athlete’s power profile. The *CP* and *W′* are derived from the fictitious athletes’ respective power profiles.

Profile	Endurance $\left(\frac{\Delta \%\mathrm{MAP}}{\Delta \log\left(T\right)}\right)$	Anaerobic Capacity (J/kg)	MAP (W/kg)	Critical Power (*CP*; W)	Anaerobic Reserve (*W′*; kJ)
Time-Trialist	−8	1400	25	304	21.5
All-Rounder	−10	1600	25	293	27.1
Sprinter	−12	1800	23	259	32.3

**Table 2:** Average power sustained over various durations for each fictitious cyclist, derived using the Péronnet–Thibault (1989) continuous exercise model.

Performance Duration	Sprinter (W)	All-Rounder (W)	Time-Trialist (W)
1 s	1251	1115	978
15 s	968	876	777
30 s	782	719	647
45 s	665	623	567
1 min	589	561	516
2 min	457	454	431
3 min	411	419	403
4 min	389	402	390
5 min	375	391	382
10 min	317	341	342
20 min	276	307	315
30 min	257	290	301
45 min	239	274	289
60 min	227	264	281
90 min	211	249	269
2 h	199	239	261
4 h	172	214	241

## Data Availability

Data and codes required to perform the simulations and analyses are available on request to the corresponding author. A public repository on GitHub will soon be available with all the material.
